# Molecular subtypes in ductal carcinoma in situ of the breast and their relation to prognosis: a population-based cohort study

**DOI:** 10.1186/1471-2407-13-512

**Published:** 2013-10-30

**Authors:** Wenjing Zhou, Karin Jirström, Rose-Marie Amini, Marie-Louise Fjällskog, Thomas Sollie, Henrik Lindman, Therese Sørlie, Carl Blomqvist, Fredrik Wärnberg

**Affiliations:** 1 Department of Surgical Science, Uppsala University, Uppsala SE-75105, Sweden; 2 Department of Clinical Sciences, Lund University, Lund, Sweden; 3 Department of Immunology, Genetics and Pathology, Uppsala University, Uppsala, Sweden; 4 Department of Oncology, Uppsala University, Uppsala, Sweden; 5 Department of Pathology, Örebro University, Örebro, Sweden; 6 Department of Genetics, Institute for Cancer Research, Oslo University Hospital, Norwegian Radium Hospital, Oslo, Norway; 7 Cancer Stem Cell Innovation Center, Oslo University Hospital, Norwegian Radium Hospital, Oslo, Norway; 8 Department of Oncology, Helsinki University Central Hospital, Helsinki, Finland

## Abstract

**Background:**

Different molecular subtypes of breast cancer have been identified based on gene expression profiling. Treatment suggestions based on an approximation of these subtypes by immunohistochemical criteria have been published by the St Gallen international expert consensus panel. Ductal carcinoma in situ (DCIS) can be classified into the same molecular subtypes. Our aim was to study the relation between these newly defined subtypes and prognosis in DCIS.

**Methods:**

TMA including 458 women from a population-based cohort with DCIS diagnosed 1986–2004 was used. Stainings for ER, PR, HER2 and Ki67 were used to classify the surrogate molecular subtypes according to the St Gallen criteria from 2011. The associations with prognosis were examined using Kaplan-Meier analyses and Cox proportional hazards regression models.

**Results:**

Surrogate molecular subtyping could be done in 381 cases. Mean follow up was 164 months. Of the classified DCIS 186 were Luminal A (48.8%), 33 Luminal B/HER2- (8.7%), 74 Luminal B/HER2+ (17.4%), 61 HER2+/ER- (16.0%) and 27 Triple Negative (7.1%). One hundred and two women had a local recurrence of which 58 were invasive. Twenty-two women had generalised disease, 8 without a prior local recurrence. We could not find a prognostic significance of the molecular subtypes other than a higher risk of developing breast cancer after more than 10 years of follow-up among women with a Triple Negative DCIS (OR 3.2; 95% CI 1.1-9.8).

**Conclusions:**

The results from this large population-based cohort, with long-term follow up failed to demonstrate a prognostic value for the surrogate molecular subtyping of DCIS using the St Gallen criteria up to ten years after diagnosis. More than ten years after diagnosis Triple Negative DCIS had an elevated risk of recurrence.

## Background

At the 12th St Gallen International Breast Cancer Conference 2011 a new classification system of biological breast cancer subtypes was adopted (Goldhirsch, [1]). The intrinsic molecular subtypes based on gene expression analyses (Perou [2], Sorlie [3]) are for practical purposes approximated using clinicopathological criteria. Systemic therapy recommendations for invasive breast cancer according to the subtype classification were also proposed. The surrogate pathology-based definitions were slightly changed at the last St Gallen conference (Goldhirsch, [4]).

Ductal carcinoma in situ (DCIS) can be classified into the same molecular subtypes as invasive breast cancer by gene expression analysis [5,6]. Immunohistochemistry (IHC) has also been used for DCIS by Livasy et al. (Livasy) to mimic the molecular subtypes. However, the new St Gallen classification has not been applied on DCIS before. The main difference between the system used by Livasy and the St Gallen criteria is the inclusion of proliferation to the classification, measured by Ki67.

A clinically useful histopathological classification system for DCIS predicting prognosis is still missing. Survival is excellent after a primary diagnosis of DCIS, but the risk of recurrence is considerably high (EBCTCG [7]). Hence, identification of biomarkers to aid in individualized treatment decisions regarding surgery and radiotherapy would be very useful. Kerlikowske et al., [8] used IHC for biomarkers including Ki67 and found that biomarkers were better than histopathological criteria for identifying risk groups for subsequent invasive cancer and Solin et al., [9] used a 21-gene array to identify risk groups after breast conserving surgery (BCS) without postoperative radiation. We have in an earlier paper shown that basal like DCIS according to the classification by Livasy et al., had a higher but not statistically significantly higher risk of recurrence [10].

In this study, our aim was to classify DCIS into the same surrogate molecular subtypes proposed by the St Gallen international expert consensus for invasive breast cancer but also to study if there was a relation between these surrogate molecular subtypes and prognosis in DCIS.

## Methods

### Patients

All women, diagnosed with a primary DCIS between 1986 and 2004 in Uppland and Västmanland, Sweden were included (n = 458). Follow-up was complete up to November 31st, 2011.

We used three primary end points; 1) “Local recurrence” - including all ipsilateral events (*in situ* and invasive), 2) “Invasive or general recurrence” - including all invasive ipsilateral events, all regional and distant metastatic events and finally 3) “All events” – including all ipsilateral events, all regional and distant metastatic events and all contralateral events. All women with an invasive ipsilateral recurrence were accordingly included as cases using both endpoint 1, 2 and 3. We did not include events occurring earlier than three months after primary diagnosis. Follow-up was divided into the first ten years and then after ten years.

### IHC and silver-enhanced in situ hybridization (SISH)

Tumour biopsies from paraffin blocks were used to construct tissue microarrays (TMA). IHC for estrogen receptor (ER), progesterone receptor (PR), human epidermal growth factor receptor 2 (HER2) and Ki67 have been performed as earlier desciribed (Zhou, Wärnberg [11]). For HER2, SISH have also been performed previously (Zhou, Wärnberg [11]).

For analysis of ER and PR, tumours with at least 1% of cell nuclei stained were considered positive, regardless of staining intensity [12]. We did all analyses with a cut of at ≥10% as well, as this is the cut off still used in Sweden. Proliferation was considered high if IHC staining for Ki67 was seen in more than 14% of tumour nuclei. We also used other cut offs for Ki67 (10% and 20%) in separate analyses. In 101 of the DCIS cases where Ki67 information was missing from the TMAs, we used an earlier scoring of Ki67 from original slides (Wärnberg [13]). However, the earlier grouping was only made into four different intervals; 0%, 1-10%, 11-30% and >30% and hence, we could not include all these cases using the 14% and 20% cut offs.

For HER2 gene amplification the American Society of Clinical Oncology/College of American Pathologists guideline and Australian HER2 Advisory Board criteria for single HER2 probe testing was used (diploid, 1 to 2.5 copies/nucleus; polysomy >2.5 to 4 copies/nucleus; equivocal, >4 to 6 copies/nucleus; low-level amplification, >6 to 10 copies/nucleus; and high-level amplification >10 copies/nucleus) and for dual HER2/CHR17 probe testing (nonamplified ratio <1.8; equivocal ratio, 1.8 to 2.2; gene amplification, >2.2). The status of HER2 expression was relying on SISH. For those cases on which SISH was missing we considered HER2 positive if the IHC score was 3+ using the HerceptTest™.

### Surrogate molecular subtypes

The different subtypes were defined and denoted by us as follows;

• **Luminal A** (ER and/or PR positive, HER2 negative and Ki67 <14%)

• **Luminal B/HER2-** (ER and/or PR positive, HER2 negative and Ki67 ≥14%),

• **Luminal B/HER2+** (ER and/or PR positive, HER2 positive),

• **HER2+/ER-** (non luminal) (ER and PR negative and HER2 positive),

• **Triple Negative** (ductal), (ER, PR and HER2 negative).

The surrogate definitions were based on the 2011 St Gallen guidelines (Goldhirsch, [1]).

Cases with missing IHC data, due to lack of tumour tissue in the TMAs, were defined as unclassified. These cases were excluded from the survival analyses.

### Statistical analyses

Baseline characteristics among patients with different molecular subtypes were compared by Chi-square for categorical variables or analysis of variance for continuous variables. Survival and probabilities of local recurrence and invasive or general disease among patients with different molecular subtypes were first compared by the Kaplan-Meier method. Cox proportional hazards regression models were used to calculate hazard ratios (HRs) with 95% confidence intervals (CIs), with adjustment for type of surgery and postoperative radiotherapy in the multivariate analysis. Data were analyzed using the SAS (SAS Institute, Cary, NC) software.

This study was approved by the Ethics Committee at Uppsala University Hospital (Dnr 2005: 118).

## Results

Three hundred and eighty-one of the 458 DCIS cases could be classified into the surrogate molecular subtypes using 1% and 14% as cut offs for hormonal receptor status and proliferation, respectively. Of the classified women 186 were Luminal A (40.6%), 33 Luminal B/HER2- (7.2%), 74 Luminal B/HER2+ (16.2%), 61 HER2+/ER- (13.3%) and 27 Triple Negative (5.9%) (Table 1). This leaves 77 (16.8%) unclassified due to missing IHC data for one or more of the biomarkers needed. When using the cut off ≥10% for the hormone receptor status the corresponding numbers were; 184 Luminal A (47.9%), 30 Luminal B/HER2- (7.8%), 64 Luminal B/HER2+ (16.7%), 71 HER2+/ER- (18.5%) and 35 Triple Negative (9.1%) and 74 unclassified (16.2%).

**Table 1:** Characteristics of DCIS by surrogate molecular subtypes according to the St Gallen international expert consensus 2011 (n=458)

**Ductal carcinoma in situ characteristics**	**All**	**Luminal A**	**Luminal B/HER2-**	**Luminal B/HER2+**	**HER2+/ER-**	**Triple negative**	**Unclassified**	**P value**^**b**^	**P value**^**c**^
	**n=458**	**n=186, (%)**	**n=33, (%)**	**n=74, (%)**	**n=61, (%)**	**n=27, (%)**	**n=77, (%)**		
Percentage of all	n=458	(40.6)	(7.2)	(16.2)	(13.3)	(5.9)	(16.8)		
Percentage of all classified	n=381	(48.8)	(8.7)	(19.4)	(16.0)	(7.1)	-		
**Age** mean, years	58.2	59.6	55.2	55.2	58.4	59.2	58.2		
< 50	121(26.4)	48 (25.8)	12 (36.4)	23 (31.1)	14 (23.0)	8 (29.6)	16 (20.8)	0.38	0.42
50- 65	198 (43.2)	77 (41.4)	14 (42.4)	33 (44.6)	30 (49.2)	7 (25.9)	37 (48.0)		
> 65	139 (30.3)	61 (32.8)	7 (21.2)	18 (24.3)	17 (27.9)	12 (44.4)	24 (31.2)		
**Detection mode**									
Screening	345 (75.5)	134 (72.0)	27 (81.8)	67 (90.5)	44 (72.1)	18 (66.7)	55 (71.4)	0.10	0.16
Clinically	112 (24.5)	51 (27.4)	6 (18.2)	7 (9.5)	17 (27.9)	9 (33.3)	22 (28.6)		
**Tumor size**									
Unifocal, mean, mm	16.7	14.9	16.5	16.4	21.8	19.9	16.3	0.93^d^	0.91^d^
Multifocal (number)	n=54	n=22	n=3	n=11	n=8	n=3	n=7		
**Histopathological grade**^**a**^									
Grade 1	37 (8.1)	23 (12.4)	1 (3.0)	1 (1.4)	1 (1.6)	1 (3.7)	10 (13.0)	<0.01	<0.01
Grade 2	203 (44.6)	121 (65.0)	17 (51.5)	20 (27.0)	6 (9.8)	9 (33.3)	32 (41.6)		
Grade 3	215 (47.3)	42 (22.6)	15 (45.5)	53 (71.6)	54 (88.5)	17 (63.0)	35 (44.4)		
**Type of surgery**									
Breast Conserving Surgery	359 (78.4)	151 (81.2)	28 (84.8)	57 (77.0)	41 (67.2)	22 (81.5)	60 (77.9)	0.17	0.27
Mastectomy	99 (21.6)	35 (18.8)	5 (15.2)	17 (23.0)	20 (32.8)	5 (18.5)	17 (22.1)		
**Postoperative radiotherapy**									
Yes	161 (35.2)	63 (33.9)	17 (51.5)	27 (36.5)	23 (37.7)	9 (33.3)	22 (28.6)	0.42	0.33
No	297 (64.8)	123 (66.1)	16 (48.5)	47 (63.5)	38 (62.3)	18 (66.7)	55 (71.4)		

^a^DCIS were classified according to the European Organization for Research and Treatment of Cancer (EORTC) system.

^b^P-values were calculated between molecular subgroups by IHC, unclassified lesions were excluded.

^c^P-values were calculated between molecular subgroups by IHC, unclassified lesions were included.

^d^Chi-square test of categorical size groups (unifocal vs. multifocal).

Baseline characteristics of the 458 DCIS are presented in Table 1. The HER2+/ER-, Luminal B/HER2+, HER2+/ER- and Triple Negative subtypes were more often grade 3 compared to Luminal A and Luminal B/HER2- tumours. Only 22.6% of Luminal A tumours were grade 3. Type of surgery, mastectomy or BCS and postoperative radiotherapy were decided according to local traditions. About 45% of women undergoing BCS received postoperative radiotherapy. No woman received endocrine or chemotherapy after primary surgery. Mean follow up was 164 months (range 3–293). Fifteen women died from breast cancer and another 96 died from other causes. One hundred and two women had an ipsilateral local recurrence of which 52 were invasive and 50 had a new DCIS. Six of the 50 ipsilateral *in situ* recurrences had first an *in situ* recurrence and then followed by a later ipsilateral invasive local recurrence. The six *in situ* events followed by an invasive event were regarded as “Local recurrences” at the time of the *in situ* event, and as “Invasive or general recurrences” at the time of the subsequent invasive event. Twenty-two women had generalized disease, 8 of those with no prior local recurrence. Mean follow-up to an invasive local recurrence was 95.1 months (range 4–280) and to an *in situ* recurrence 53.3 months (10–244). Forty-five women had a contralateral breast cancer. Eleven of these were secondary to an ipsilateral event. Six of the 45 had a contralateral invasive cancer and then developed generalized disease. These six women were censored at the time of the contralateral cancer event in the survival analyses for “Local recurrence” and “Invasive or general recurrence”.

With the Luminal A subtype as reference, Cox regression analyses showed no statistically significant differences between subtypes regarding “Local recurrence” or “Invasive or general recurrence” (Table 2). However, all subtypes showed a non-significantly higher risk of “Local recurrence” compared to Luminal A during the first ten years after diagnosis and treatment. We also looked at “Local recurrence” risk after ten years of follow-up. Even if the numbers were small and no statistically significant differences were seen, notably the HER2+/ER- subtype had the highest risk during the first ten years and the lowest risk after ten years, compared with the other surrogate molecular subtypes (HR 1.77, CI 95%; 0.85-3.68 and HR 0.58; 0.06-5.89). Compared to the highest risk of “Local recurrence” for the HER2+/ER- subtype during the first ten years the risk for an “Invasive or general recurrence” was the lowest (HR 0.98 CI 95%; 0.31-3.17) compared to the reference subtype Luminal A. The Luminal B/HER2-, Luminal B/HER2+ and Triple Negative subtypes had about twice as high risk, but this was not statistically significant. Looking at “All events” we could not find any statistically significant differences between the surrogate molecular subtypes during the first ten years of follow-up. After ten years however, the risk of any event was lower, but not statistically significant lower, in the Luminal B/HER2+ and HER2+/ER- subtypes (HR 0.39, CI 95%; 0.11-1.45 and HR 0.20; 0.03-1.58 respectively) while the risk was statistically significantly higher in the Triple Negative subtype (HR 3.21, 95% CI; 1.05-9.83). All analyses were done for all women and for all women treated with BCS separately and as results looked similar data are not shown.

**Table 2:** Cox regression analyses of survival among surrogate molecular subtypes by immunohistochemistry in primary DCIS (n=458), by follow-up period

	**Follow-up period**			
	**> 3 months – 10 years**		**> 10 years**	
**Type of event**	**Unadjusted HR (95% CI)**	Adjusted* HR (95% CI)	**Unadjusted HR (95% CI)**	Adjusted* HR (95% CI)
**Local recurrence (*in situ *** **or invasive)**	No. of events: 84		No. of events: 17	
Luminial A	1.0 (reference)	1.0 (reference)	1.0 (reference)	1.0 (reference)
Luminal B/HER2-	1.39 (0.64-3.01)	1.61 (0.73-3.54)	no events	-
Luminal B/HER2+	1.31 (0.72-2.40)	1.63 (0.84-3.17)	0.78 (0.21-2.89)	1.01 (0.22-4.62)
HER2+/ER-	1.21 (0.62-2.34)	1.77 (0.85-3.68)	0.27 (0.03-2.14)	0.58 (0.06-5.89)
Triple negative	1.37 (0.57-3.28)	1.38 (0.56-3.38)	0.94 (0.12-7.44)	0.78 (0.09-7.22)
Unclassified	0.73 (0.36-1.49)	0.77 (0.38-1.58)	0.95 (0.25-3.60)	1.19 (0.29-4.78)
**Invasive or general recurrence**	No. of events: 47		No. of events: 19	
Luminial A	1.0	1.0	1.0	1.0
Luminal B/HER2-	2.02 (0.80-5.13)	2.51 (0.97-6.49)	no events	-
Luminal B/HER2+	1.49 (0.68-3.25)	1.97 (0.83-4.67)	0.78 (0.25-2.44)	0.72 (0.21-2.56)
HER2+/ER-	0.71 (0.24-2.12)	0.98 (0.31-3.17)	no events	-
Triple negative	2.24 (0.83-6.06)	1.99 (0.70-5.63)	0.82 (0.11-6.35)	0.63 (0.07-5.48)
Unclassified	0.70 (0.26-1.90)	0.84 (0.31-2.33)	0.54 (0.12-2.42)	0.53 (0.12-2.42)
**All events**	No. of events: 112		No. of events: 28	
Luminial A	1.0	1.0	1.0	1.0
Luminal B/HER2-	1.24 (0.62-2.46)	1.47 (0.73-2.94)	no events	-
Luminal B/HER2+	1.20 (0.71-2.01)	1.53 (0.87-2.71)	0.40 (0.12-1.37)	0.39 (0.11-1.45)
HER2+/ER-	0.92 (0.49-1.70)	1.28 (0.65-2.52)	0.17 (0.02-1.25)	0.20 (0.03-1.58)
Triple negative	1.30 (0.61-2.75)	1.37 (0.64-2.98)	2.95 (1.07-8.16)	3.21 (1.05-9.83)
Unclassified	0.78 (0.43-1.39)	0.82 (0.45-1.48)	0.85 (0.28-2.58)	1.02 (0.33-3.21)

*Adjusted for age, mode of detection, size, grade, surgery and radiotherapy.

**Figure 1:**
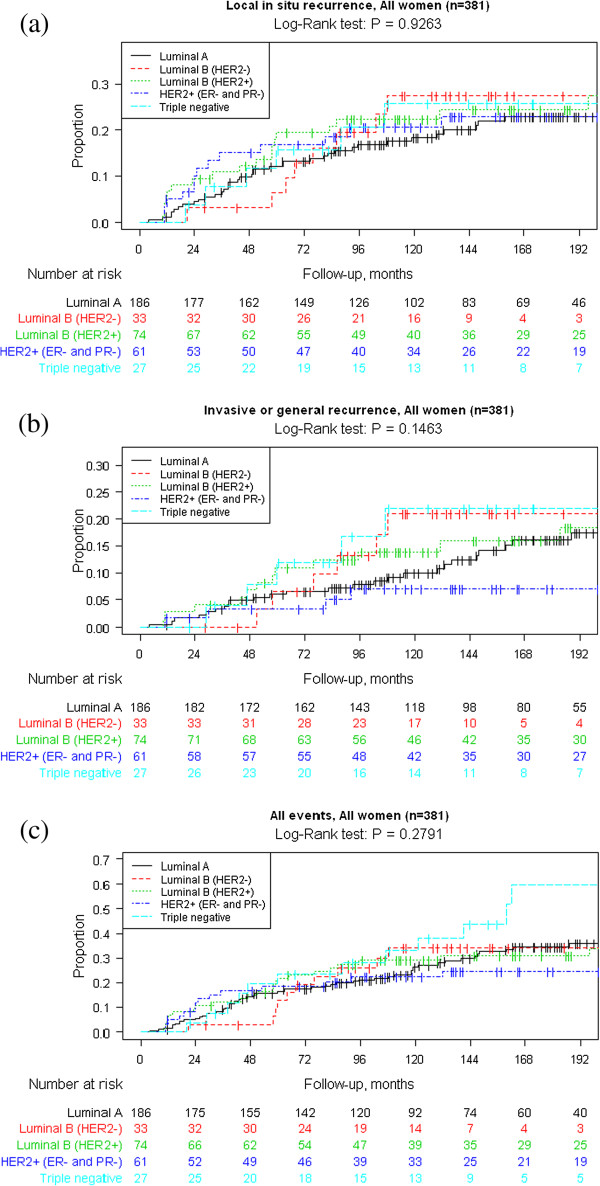
Kaplan–Meier analyses of a) local recurrence, b) invasive or general recurrence and c) all events by DCIS molecular subtypes by immunohistochemistry according to St Gallen criteria in 381 women with a primary DCIS.

**Figure 2:**
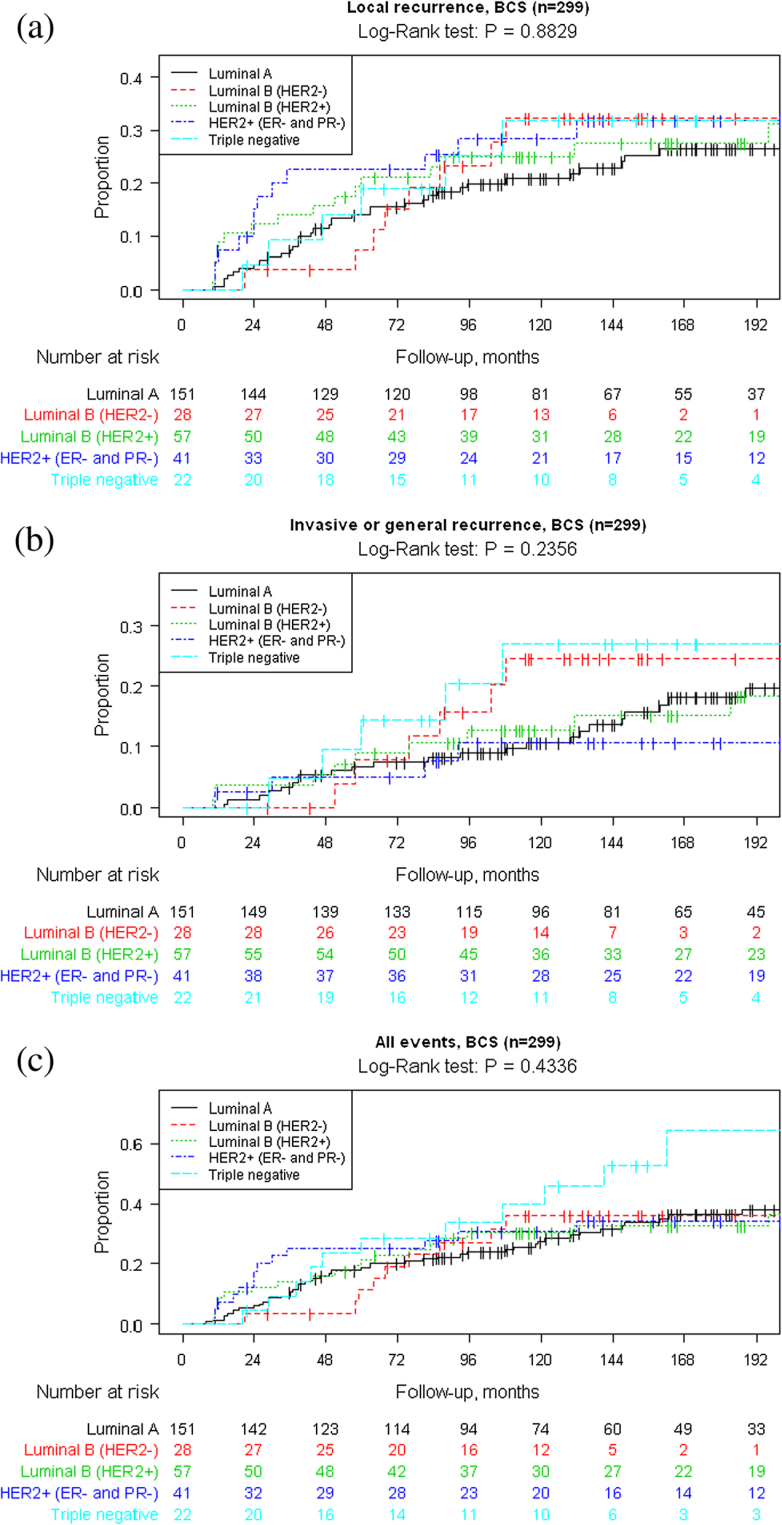
Kaplan–Meier analyses of a) local recurrence, b) invasive or general recurrence and c) all events by DCIS molecular subtypes by immunohistochemistry according to St Gallen criteria in 300 women with a primary DCIS undergoing breast-conserving surgery (BCS).

The Kaplan-Meier survival analyses are presented in Figures 1 and 2. Data are shown for all women with a primary DCIS and separately for those women undergoing BCS. Graphs are presented for a) “Local recurrence”, b) “Invasive or general recurrence” and c) “All events”. We could not see any statistically significant differences between the surrogate molecular subtypes in any of the analyses.

All results were similar when using the different cut-offs for Ki67 (10%, 14% and 20%) (data not shown). All analyses were also done with the cut off ≥10% for ER positivity. Results were not substantially different and data are not shown. We performed separate analyses stratified by post-operative radiotherapy in women undergoing BCS with similar results (data not shown).

## Discussion

In this large population-based cohort of DCIS with almost 14 years of follow-up we classified the tumours as proposed by the St Gallen international expert consensus panel for invasive breast cancer, 2011 (Goldhirsch). Despite more than 100 local recurrences and almost 70 invasive events we found very sparse prognostic information using the intrinsic surrogate molecular subtype classification. Based on few events, we found a higher risk for “All events” for the Triple Negative subtype after ten years of follow-up. Interestingly, the HER2+/ER- subtype was associated with a higher risk of local recurrence but a lower risk for invasive recurrence compared with the two Luminal B subtypes and Triple Negative tumours.

This is a retrospective study where treatment decisions were based on information from the DCIS tumours. During this period IHC was not routinely performed on pure DCIS cases. ER, PR and HER2 status was not available and molecular subtypes were not taken into consideration. When studying prognosis for the different subtypes in this study, we adjusted for the type of surgery and for post-operative radiotherapy as this is known to effect recurrence risk.

There is no clinically established classification of DCIS that helps us predicting the prognosis for an individual woman. The most common grading system used today is nuclear grade. High grade and large size has been shown to be of some prognostic relevance for local recurrence (EBCTCG) but we lack factors that predict risk for developing invasive cancer. In invasive cancer, molecular subtype has been shown to predict prognosis (Su [14], Normanno [15]) but very little data has been published regarding DCIS [10,16]. There are no publications using the proposed criteria from St Gallen, 2011(Goldhirsch, [1]) in DCIS.

HER2 status is a known risk factor for recurrence in both invasive breast carcinoma and DCIS. Two recent publications have shown an increased risk of non-invasive recurrence in HER2+ tumours [8,17]. In the study by Rakovitch et al., the combination of HER2+ and high proliferation conferred an even higher risk of non-invasive recurrence and in the study by Kerlikowske et al., the combination of HER2+, ER- and high proliferation was associated with a six times increased risk of non-invasive recurrence. Our data did go in the same direction with a higher risk for local recurrence in the HER2+/ER- subtype including both *in situ* and invasive events and, a lower risk for any invasive recurrence. Other biological markers have also been studied but there were no significant associations found between a variety of biologic markers and the risk of recurrence after a primary DCIS as reviewed by Lari and Kuerer [18].

In this study we wanted to examine whether different cut offs for Ki67 assessment could influence the prognostic ability of the Luminal A and Luminal B/HER2- molecular subtypes. The St Gallen criteria use a cut off at 14%. This cut off has been rejected by the IMPAKT working group [19] and there are also difficulties in reproducibility when scoring Ki67 [20]. By using different cut offs, tumours from a number of women will potentially change molecular subtype. In this cohort, only 10 women changed from Luminal B/HER2- to Luminal A by raising the Ki67 cut off from 14% to 20%. No women changed molecular subtype by lowering the cut off to 10%. If we had used yet another cut off, e.g., 30%, only an additional three women would have changed from Luminal B/HER2- to Luminal A. Altogether, we could not see any influence on prognosis in any of our analyses using the different Ki67 cut off levels.

If we compare the surrogate molecular subtypes using the St Gallen criteria with subtypes using the Livasy (Livasy [21]) classification, proliferation is the main difference. PR status is not used and EGFR + or CK5/6+ is necessary for defining the basal like subtype in the Livasy classification. E.g., of the 27 Triple Negative cases by the St Gallen criteria, eight were unclassified according to Livasy criteria as either EGFR or CK5/6 were missing. And, of the 35 basal like cases by Livasy, three were unclassified, 4 were Luminal A and one Luminal B/HER2- using the St Gallen criteria due to PR status. These circumstances make comparisons between studies using different criteria difficult.

## Conclusions

We could not find that the surrogate molecular subtyping proposed by the St Gallen international expert consensus for invasive breast cancer, 2011, was a prognostic useful tool in DCIS. We found a significantly higher risk of developing a new breast cancer event after ten years in the Triple Negative subtype but this was based on few events. Our data, however not statistically significant, did support newly published data indicating that HER2+ in itself is a risk factor for recurrence, but more specifically, non-invasive recurrence and this need to be further explore.

## Abbreviations

DCIS: Ductal carcinoma *in situ*; IHC: Immunohistochemistry; CI: Confidence interval; HR: Hazard ratio; TMA: Tissue microarrays; ER: Estrogen receptor; PR: Progesterone receptor; HER2: human epidermal growth factor receptor 2; CK5/6: Cytokeratin 5/6; EGFR: Epidermal growth factor receptor.

## Competing interests

The authors declare that they have no competing interests.

## Authors’ contributions

FW designed the overall study, compiled and curated the datasets, coordinated the study and helped to draft and finalize the manuscript. WZ was responsible for data analyses, manuscript preparation and editing. KJ performed IHC and SISH stainings from the TMAs, and helped to provide expertise in breast cancer pathology. WZ and RMA were involved in pathology review, scoring of stains and contributed substantially to manuscript editing. CB, MLF, HL TS and TS helped with the interpretation of the results and with drafting the manuscript. All authors have read and approved the final manuscript.

## Pre-publication history

The pre-publication history for this paper can be accessed here:

http://www.biomedcentral.com/1471-2407/13/512/prepub
